# *PON1* rs662, rs854560 and *TRIB1* rs17321515, rs2954029 Gene Polymorphisms Are Associated with Lipid Parameters in Patients with Unstable Angina

**DOI:** 10.3390/genes15070871

**Published:** 2024-07-02

**Authors:** Damian Malinowski, Krzysztof Safranow, Andrzej Pawlik

**Affiliations:** 1 Department of Pharmacokinetics and Therapeutic Drug Monitoring, Pomeranian Medical University, 70-111 Szczecin, Poland; damian.malinowski@pum.edu.pl; 2 Department of Biochemistry and Medical Chemistry, Pomeranian Medical University, 70-111 Szczecin, Poland; chrissaf@mp.pl; 3 Department of Physiology, Pomeranian Medical University, 70-111 Szczecin, Poland

**Keywords:** PON1, TRIB1, SNP, unstable angina

## Abstract

Acute coronary heart disease (CHD) is mainly caused by the rupture of an unstable atherosclerotic plaque. Many different factors can cause stenosis or even occlusion of the coronary artery lumen, such as vasculitis and platelet aggregation. Our study was performed to assess the association between *PON1* rs662, rs854560 and *TRIB1* rs17321515, rs2954029 polymorphisms and the risk of CHD, as well as the association between studied polymorphisms and selected clinical parameters affecting the risk of developing ischemic heart disease. A total of 232 patients with unstable angina were enrolled in this study. There were no statistically significant differences in the *PON1* rs662, rs854560 and *TRIB1* rs17321515, rs2954029 polymorphism distributions between the total study and control groups. Total cholesterol plasma levels were significantly higher in patients with the *PON1* rs662 TT genotype compared to those with the CC+TC genotypes, as well as in patients with the *PON1* rs854560 TT genotype compared to those with the AA+AT genotypes. LDL plasma levels were significantly increased in patients with the *PON1* rs854560 TT genotype compared to those with the AA+AT genotypes. Plasma levels of HDL were significantly decreased in patients with the *TRIB1* rs17321515 AA+AG genotypes compared to those with the GG genotype, as well as in patients with the *TRIB1* rs2954029 AA+AT genotypes compared to those with the TT genotype. Our results suggest that the analysed polymorphisms are not risk factors for unstable angina in the Polish population. However, the results of this study indicate an association between the *PON1* rs662, rs854560 and *TRIB1* rs17321515, rs2954029 polymorphisms with lipid parameters in patients with coronary artery disease.

## 1. Introduction

The main cause of the development of coronary heart disease is the atherosclerotic process that develops in the coronary arteries. Underlying the development of atherosclerotic lesions is an inflammatory process occurring in the centre of the vessel, with lipid compounds deposited therein [1]. One of the causes of atherosclerosis development is disorders of lipid metabolism. The association of lipid disorders with some genetic loci has now been established [2,3,4,5]. Among the genes affecting lipid metabolism are *PON1*, encoding Paraoxonase 1, and *TRIB1*, encoding the serine/threonine kinase-like protein tribbles homolog 1.

PON1 is a glycoprotein aryldialkylphosphatase with a mass of 43–45 kDa and a length of 354–355 amino acids. Previous studies have shown that this protein exerts a protective effect on blood vessels by inhibiting the development of the atherosclerotic process through its anti-inflammatory activity. It also inhibits platelet adhesion and aggregation, thereby preventing the formation of thrombi and embolisms [6]. The protein also exhibits antioxidant and anti-apoptotic properties and has a beneficial effect on lipid metabolism by increasing cholesterol excretion, inhibiting lipid oxidation in both low-density lipoproteins (LDL) and high-density lipoproteins (HDL) and inhibiting lipoprotein peroxidation [7].

The *TRIB1* gene encodes the serine/threonine kinase-like protein tribbles homolog 1, an adaptor protein that regulates numerous metabolic pathways [8]. TRIB1 protein has been detected in many tissues, including the coronary arteries, especially in patients with advanced atherosclerosis and ischemic disease. The protein is also a known regulator of lipid synthesis. In an animal model, blocking TRIB1 increased plasma triglyceride (TG) and cholesterol levels, whereas its administration increased the number of low-density lipoprotein receptors (LDLRs) and decreased plasma LDL-C levels [9,10]. Numerous clinical studies have shown an association between polymorphisms of the *PON1* and *TRIB1* genes and various cardiovascular diseases [11,12,13,14]. The effects of these genes on lipid parameters have also been demonstrated [14,15].

Several polymorphisms have been detected within the *PON1* and *TRIB1* genes, which may alter their expression and subsequently affect protein synthesis. Previous studies have shown that these polymorphisms can affect lipid metabolism and are risk factors for the development of various cardiovascular diseases [14,15]. Among these polymorphisms are *PON1* rs662 and rs854560 and *TRIB1* rs17321515 and rs2954029. This study aimed to evaluate the association of these polymorphisms with the risk of coronary artery disease, specifically unstable angina, and to investigate their relationship with biochemical parameters in these patients.

## 2. Materials and Methods

### 2.1. Study Subjects

A case–control study was conducted. A total of 232 patients (mean age 62.07 ± 9.68 years; 172 male, 60 female) with coronary artery disease, classified as unstable angina based on coronary angiography and treated in the Department of Cardiology, were enrolled in this study. The diagnosis of unstable angina was based on the typical clinical presentation and confirmation of significant coronary artery lumen stenosis (>70%) during coronary angiography. Patients with a definitive diagnosis of myocardial infarction based on a significant increase in the markers of myocardial damage (troponin T and myoglobin), autoimmune diseases or cancer were excluded from this study.

The control group consisted of 144 patients (mean age 67.4 ± 10.6 years; 54 male, 90 female) who underwent coronary angiography for unexplained chest pain. In this group of patients, coronary angiography showed no coronary lumen stenosis. The exclusion criteria included a history of inflammatory disease or cancer. Arterial hypertension was diagnosed in 62.5% of patients with unstable angina and 39.6% of controls. Diabetes was diagnosed in 24.6% of patients and 6.3% of controls.

The biochemical parameters (triglycerides, total cholesterol, high-density lipoprotein cholesterol (HDL) and low-density lipoprotein cholesterol (LDL)), as well as anthropogenic parameters (age, weight and height), were collected from both studied groups. The study protocol was approved by the Ethics Committee of the Pomeranian Medical University, Szczecin, Poland under registry number KB-0012/46/17. Written informed consent was obtained from all subjects.

### 2.2. Methods

Peripheral venous blood samples were collected from each subject into tubes containing EDTA during routine check-ups. The blood samples were stored at −80 °C until DNA extraction. Genomic DNA was extracted from peripheral blood leucocytes using a Genomic Mini AX Blood 1000 Spin kit (A&A Biotechnology, Gdynia, Poland), following the manufacturer’s protocol. The DNA was diluted to equal concentrations of 20 ng/µL, based on spectrophotometric absorbance measurements (260/280 nm) using a DeNovix DS-11 FX+ Spectrophotometer/Fluorometer (Wilmington, DE, USA). The DNA samples were stored at −80 °C prior to genotyping analysis.

### 2.3. Genotyping

Genotyping was performed for the following single nucleotide polymorphisms (SNPs): *PON1* rs662, rs854560 and *TRIB1* rs17321515, rs2954029 (TaqMan Assay IDs: rs662: C___2548962_20, rs854560: C___2259750_20, rs17321515: C__33068431_10, rs2954029: C__15954645_10; Life Technologies, Waltham, MA, USA). Genotyping was performed using pre-validated allelic discrimination TaqMan real-time PCR assays (containing VIC^®^ and FAM™ fluorochromes probes and two specific primers for each of the SNP variants), nuclease-free water and TaqMan GTXpress Master Mix (Life Technologies, Waltham, MA, USA) (Table 1).

All reactions were run in a final volume of 12 µL (in duplicates) with the following reaction temperature profile: 95 °C for 20 s, followed by 40 cycles of 95 °C for 1 s and 60 °C for 20 s.

Genotyping was conducted in a ViiA7 Real-Time PCR System (Applied Biosystems, Waltham, MA, USA). Genotypes were assigned using TaqMan Genotyper software v.1.3 (Thermo Fisher Scientific, Waltham, MA, USA). A randomly selected 20% of the analysed samples were repeated as internal quality controls. The Results and Discussion paragraphs show the genotypes/alleles captured using real-time PCR.

### 2.4. Statistical Analysis

Genotype distributions with Hardy–Weinberg equilibrium (HWE) were assessed using the Fisher’s exact test. Odds ratios (ORs) and 95% confidence intervals (95% CIs) were calculated using continuity-corrected Wald intervals. For the demographic data, alignment with normal distribution was assessed using the Shapiro–Wilk test, and further analyses were performed using either a one-way parametric ANOVA test or a one-way non-parametric ANOVA test (Kruskal–Wallis test). The χ2 test was used to compare the distributions of genotypes and alleles between the groups. The distribution of quantitative clinical parameters in the study group differed significantly from a normal distribution (Shapiro–Wilk test). Therefore, these parameters were compared between groups using the non-parametric Mann–Whitney test. A value of *p* < 0.05 was considered statistically significant without correction for multiple testing. The analyses were performed using Statistica ver. 13.2 software (TIBCO Software Inc., Tulsa, OK, USA).

## 3. Results

The distribution of *PON1* rs662, rs854560 and *TRIB1* rs17321515, rs2954029 genotypes met HWE and is presented in Table 2.

There were no statistically significant differences in the distribution of the *PON1* rs662, rs854560 genotypes and alleles between patients with unstable angina and control subjects (Table 2). There were also no statistically significant differences in the distribution of these genotypes and alleles between patients with and without diabetes or between those with and without arterial hypertension (Table 3 and Table 4).

There were also no statistically significant differences in the distribution of *TRIB1* rs17321515, rs2954029 genotypes and alleles between patients with unstable angina and the control subjects (Table 2), between patients with and without diabetes or between patients with and without arterial hypertension (Table 3 and Table 4).

We also analysed the associations between the studied genotypes and biochemical parameters in patients with unstable angina (Table 5, Table 6, Table 7 and Table 8).

Total cholesterol plasma levels were significantly higher in patients with the *PON1* rs662 TT genotype compared to those with the CC+TC genotypes (*p* = 0.03), as well as in patients with the *PON1* rs854560 TT genotype compared to those with the AA+AT genotypes (*p* = 0.003) (Table 5 and Table 6). LDL plasma levels were significantly increased in patients with the *PON1* rs854560 TT genotype compared to those with the AA+AT genotypes (*p* = 0.017) (Table 6).

Plasma levels of HDL were significantly decreased in patients with the *TRIB1* rs17321515 AA+AG genotypes compared to those with the GG genotype, as well as in patients with the *TRIB1* rs2954029 AA+AT genotypes compared to those with the TT genotype (Table 7 and Table 8).

## 4. Discussion

Underlying the development of ischemic heart disease and acute coronary syndromes are a multitude of causes, among which the atherosclerotic process and ongoing inflammation in the vessels play key roles. Among other components, the atherosclerotic plaque consists of lipids, and abnormal lipid metabolism is one important risk factor for coronary artery disease. Studies have shown that lipid metabolism is regulated by several genes, which can influence the development of coronary artery disease [2,3]. The purpose of the present study was to evaluate the association of the *PON1* rs662, rs854560 and *TRIB1* rs17321515, rs2954029 gene polymorphisms with the risk of coronary artery disease in the form of unstable angina and to investigate their association with biochemical parameters in these patients. Our results showed no statistically significant differences in the distribution of the genotypes studied between the group of patients with unstable angina and the control subjects. Instead, we showed associations between the polymorphisms studied and some lipid parameters.

To date, polymorphisms in the *PON1* gene have been studied in relation to lipid parameters and various cardiovascular diseases. Some of the more frequently studied polymorphisms in the *PON1* gene are the rs662 and rs854560 polymorphisms. The missense mutation rs622 (c.575A>G, in our study the complementary strand was analysed, thus T>C in the results section) is caused by the conversion of glutamine to arginine at position 192 (p.Gln192Arg) and affects paraoxonase activity [16]. Previous studies have demonstrated associations between this polymorphism and lipid parameters [17]. The *PON1* rs622 TT genotype was associated with decreased PON1 expression and a significantly higher Ox-LDL/apoB ratio [18]. Increased Ox-LDL/apoB concentrations may be due to reduced serum antioxidant capacity, including reduced PON1 action. The results of our study show that total cholesterol plasma levels were significantly higher in patients with the *PON1* rs662 TT genotype. Li et al. investigated the association between the PON1 rs662 polymorphism and serum lipid levels and human longevity in the Bama Zhuang population. The authors showed that the frequency of the rs662 T allele was significantly higher in the longevity group than in the control group. Triglyceride levels were lower in subjects with the TT genotype, whereas total cholesterol and HDL-C levels were lower in subjects with the CC genotype [19].

The missense mutation rs854560 (c.163A>T) is caused by the conversion of leucine to methionine at position 55 (p.Leu55Met). The *PON1* rs854560 TT genotype is associated with decreased PON1 concentration and activity [16,18]. Previous studies have demonstrated associations between this polymorphism and lipid parameters. A meta-analysis conducted by Luo et al. suggested that the *PON1* rs854560 polymorphism is associated with HDL plasma levels in Caucasians and subjects of other ethnic origins [20]. The *PON1* rs854560 TT genotype was also associated with dyslipidaemia and cardiovascular diseases, as well as mortality in haemodialysis patients [21].

In our study, total cholesterol plasma levels were significantly higher in patients with the *PON1* rs662 TT genotype compared to those with the CC+TC genotypes, as well as in patients with the *PON1* rs854560 TT genotype compared to those with the AA+AT genotypes. LDL plasma levels were significantly increased in patients with the *PON1* rs854560 TT genotype compared to those with the AA+AT genotypes. The elevated total cholesterol and LDL plasma concentrations observed in patients with the TT genotype could be explained by the reduced expression of paraoxonase 1 and the less positive effects of this protein on lipid metabolism [21].

Previous studies suggest associations between *PON1* gene polymorphisms and various forms of cardiovascular disease, such as coronary artery disease, myocardial infarction and ischemic stroke, in different populations [11,12,21]. However, the results varied among the populations studied. A meta-analysis conducted by Ashiq et al. suggested that the *PON1* gene rs662 polymorphism was significantly associated with coronary artery disease; however, the rs854560 polymorphism was not significantly associated with the disease [22]. A meta-analysis conducted by Zeng et al. indicated that the *PON1* gene rs854560 polymorphism is associated with the risk of ischemic stroke and the rs662 polymorphism is associated with susceptibility to coronary artery disease [23]. A meta-analysis conducted by Deng et al. suggested that the *PON1* gene rs662 polymorphism is associated with coronary artery disease risk in the Chinese population [24].

The *TRIB1* gene encodes a serine/threonine kinase-like protein that plays a regulatory role in lipid metabolism. Blocking the *TRIB1* gene in mice increased plasma TG and plasma cholesterol levels [25]. In contrast, increasing the expression of this gene resulted in lower plasma lipid levels in mice [9]. In addition, it has been observed that an increase in *TRIB1* gene expression causes a decrease in plasma levels of proprotein convertase subtilisin/kexin type 9 (PCSK9), an increase in LDLR density and a decrease in LDL-C levels [26]. In addition, deletion of the *TRIB1* gene has been shown to reduce LDLRs and raise plasma LDL-C levels [10]. The polymorphisms rs2954029 and rs17321515 of the *TRIB1* gene have been shown to alter the secondary structure of mRNA [27], thereby affecting TRIB1 protein expression [9,28]. Previous studies have demonstrated associations between the polymorphisms rs2954029 and rs17321515 of the *TRIB1* gene and lipid parameters. Previous studies have shown that *TRIB1* gene polymorphisms are associated with TG levels [2,29,30,31,32,33,34]. Other studies have indicated that the rs17321515 polymorphism in the *TRIB1* gene is associated with plasma total cholesterol levels [35]. Patients with the A allele had increased levels of plasma total cholesterol. A meta-analysis conducted by Wei et al. indicated associations between polymorphisms rs2954029 and rs17321515 of the *TRIB1* gene and the levels of total cholesterol and LDL cholesterol in plasma [14]. Patients with the rs17321515 and rs2954029 A alleles had increased levels of low-density lipoprotein cholesterol and total cholesterol. The rs2954029 and rs17321515 polymorphisms of the *TRIB1* gene were studied in patients with circulatory system diseases. Associations between the rs2954029 and rs17321515 polymorphisms and coronary artery disease were demonstrated. A meta-analysis conducted by Jiang et al. found an association between the rs2954029 A allele and an increased risk of coronary artery disease [13]. A meta-analysis conducted by Wei et al. found associations between rs2954029 and rs17321515 and coronary artery disease in the Asian population [14]. Ollila et al. demonstrated associations between the *TRIB1* rs17321515 G allele and *TRIB1* rs2954029 T allele and blood total cholesterol levels [36]. Varbo et al. demonstrated that *TRIB1* rs2954029 TA+AA genotypes were associated with increased levels of triglycerides, total cholesterol, apolipoprotein B, low-density lipoprotein cholesterol and reduced levels of high-density lipoprotein cholesterol [37]. Ikeoka et al. indicated that in women, the *TRIB1* rs2954029 AA genotype was significantly associated with increased triglyceride levels [38]. Our results showed significantly reduced plasma HDL levels in patients with *TRIB1* rs17321515 AA+AG genotypes compared to patients with the GG genotype, and in patients with *TRIB1* rs2954029 AA+AT genotypes compared to patients with the TT genotype.

Additionally, the results of our study showed no associations between the *PON1* rs662, rs854560 and *TRIB1* rs17321515, rs2954029 polymorphisms and an increased risk of coronary artery disease in the form of unstable angina in our population.

Coronary artery disease is caused by numerous factors affecting lipid parameters, coagulation and platelet aggregation processes and the development of inflammation in blood vessels. Several genetic polymorphisms are associated with an increased risk of developing coronary artery disease [3]. Due to the complexity of the development of coronary artery disease, it seems that the influence of individual polymorphisms on its occurrence is small. The contribution of genetic polymorphisms to the development of ischemic disease must be considered along with other factors affecting its pathogenesis. Demonstrating the impact of genetic polymorphisms on the risk of ischemic disease often requires multicentre GWAS studies involving a significant number of patients. However, the value of a case–control study may lie in demonstrating associations between the polymorphisms studied and some clinical and biochemical parameters, which may prompt further research. In our study, we did not demonstrate statistically significant associations between *PON1* rs662, rs854560 and *TRIB1* rs17321515, rs2954029 polymorphisms and the risk of coronary artery disease in the form of unstable angina in our population. In contrast, we demonstrated associations between the polymorphisms studied and lipid parameters. A limitation of our study is the small number of cases included. We cannot exclude the possibility that with a much larger study group, we could have obtained statistically significant associations between the polymorphisms studied and unstable angina.

## 5. Conclusions

The results of this study suggest associations between the *PON1* rs662, rs854560 and *TRIB1* rs17321515, rs2954029 polymorphisms and lipid parameters in patients with coronary artery disease.

## Figures and Tables

**Table 1:** TaqMan assays used for study purposes and SNP nomenclature.

SNP ID *	Legacy Nomenclature	Name Alias	SNP Variant	TaqMan ID	VIC^®^	FAM™
rs662	c.575A>G	p.Gln192Arg	missense	C___2548962_20	C	T
rs854560	c.163A>T	p.Leu55Met	missense	C___2259750_20	A	T
rs17321515	-	-	unknown	C__33068431_10	A	G
rs2954029	-	-	unknown	C__15954645_10	A	T

* reference SNP ID notation assigned by dbSNP.

**Table 2:** Distribution of *PON1* rs662, rs854560 and *TRIB1* rs17321515, rs2954029 genotypes and alleles in patients with unstable angina and controls.

	Control Group (*n* = 144)		Unstable Angina (*n* = 232)		*p*-Value ^^^	Compared Genotypes or Alleles	*p*-Value ^#^	OR (95% CI)
	n	%	n	%				
***PON1*** **rs662**								
**genotype**								
TT	79	54.86%	127	54.74%	0.818	CC+TC vs. TT	1.00	1.01 (0.66–1.53)
TC	52	36.11%	88	37.93%		CC vs. TC+TT	0.56	0.80 (0.38–1.69)
CC	13	9.03%	17	7.33%		CC vs. TT	0.69	0.81 (0.38–1.77)
						TC vs. TT	0.91	1.05 (0.68–1.64)
						CC vs. TC	0.54	0.77 (0.35–1.72)
**Allele**								
T	210	72.92%	342	73.71%				
C	78	27.08%	122	26.29%		C vs. T	0.87	0.96 (0.69–1.34)
***PON1*** **rs854560**								
**genotype**								
AA	63	43.75%	99	42.67%		TT+AT vs. AA	0.92	1.05 (0.69–1.59)
AT	64	44.44%	108	46.55%	0.907	TT vs. AT+AA	0.74	0.90 (0.47–1.74)
TT	17	11.81%	25	10.78%		TT vs. AA	0.86	0.94 (0.47–1.87)
						AT vs. AA	0.82	1.07 (0.69–1.67)
						TT vs. AT	0.73	0.87 (0.44–1.74)
**Allele**								
A	190	65.97%	306	65.95%				
T	98	34.03%	158	34.05%		T vs. A	1.00	1.00 (0.73–1.37)
***TRIB1*** **rs17321515**								
**genotype**								
AA	34	23.61%	59	25.43%		GG+AG vs. AA	0.71	0.91 (0.56–1.47)
AG	79	54.86%	109	46.98%	0.285	GG vs. AG+AA	0.22	1.39 (0.85–2.27)
GG	31	21.53%	64	27.59%		GG vs. AA	0.65	1.19 (0.65–2.17)
						AG vs. AA	0.44	0.80 (0.48–1.33)
						GG vs. AG	0.16	1.50 (0.89–2.51)
**Allele**								
A	147	51.04%	227	48.92%				
G	141	48.96%	237	51.08%		G vs. A	0.60	1.09 (0.81–1.46)
***TRIB1*** **rs2954029**								
**genotype**								
AA	34	23.61%	61	26.29%		TT+AT vs. AA	0.63	0.87 (0.54–1.41)
AT	80	55.56%	109	46.98%	0.245	TT vs. AT+AA	0.22	1.39 (0.84–2.28)
TT	30	20.83%	62	26.73%		TT vs. AA	0.76	1.15 (0.63–2.11)
						AT vs. AA	0.31	0.76 (0.46–1.26)
						TT vs. AT	0.12	1.52 (0.90–2.56)
**Allele**								
A	148	51.39%	231	49.78%				
T	140	48.61%	233	50.22%		T vs. A	0.71	1.07 (0.80–1.43)

^ χ^2^ test. ^#^ Fisher’s exact test. HWE: control group *p* = 0.304, unstable angina *p* = 0.745 for *PON1* rs662. HWE: control group *p* = 0.904, unstable angina *p* = 0.578 for *PON1* rs854560. HWE: control group *p* = 0.241, unstable angina *p* = 0.362 for *TRIB1* rs17321515. HWE: control group *p* = 0.179, unstable angina *p* = 0.358 for *TRIB1* rs2954029.

**Table 3:** Distribution of the *PON1* rs662, rs854560 genotypes and alleles in unstable angina patients with and without diabetes mellitus (DM).

	Without Diabetes Mellitus (*n* = 175)		Diabetes Mellitus (*n* = 57)		*p*-Value ^^^	Compared Genotypes or Alleles	*p*-Value *	OR (95% CI)
	n	%	n	%				
***PON1*** **rs662**								
**genotype**								
TT	92	52.57%	35	61.41%	0.479	CC+TC vs. TT	0.29	0.70 (0.38–1.28)
TC	69	39.43%	19	33.33%		CC vs. TC+TT	0.77	0.64 (0.18–2.31)
CC	14	8.00%	3	5.26%		CC vs. TT	0.56	0.56 (0.15–2.08)
						TC vs. TT	0.34	0.72 (0.38–1.37)
						CC vs. TC	1.00	0.78 (0.20–2.99)
**Allele**								
T	253	72.29%	89	78.07%				
C	97	27.71%	25	21.93%		C vs. T	0.27	0.73 (0.44–1.21)
***PON1*** **rs854560**								
**genotype**								
AA	78	44.57%	21	36.84%		TT+AT vs. AA	0.36	1.38 (0.75–2.55)
AT	77	44.00%	31	54.39%	0.391	TT vs. AT+AA	0.81	0.75 (0.27–2.09)
TT	20	11.43%	5	8.77%		TT vs. AA	1.00	0.93 (0.31–2.77)
						AT vs. AA	0.26	1.50 (0.79–2.83)
						TT vs. AT	0.46	0.62 (0.21–1.80)
**Allele**								
A	233	66.57%	73	64.04%				
T	117	33.43%	41	35.96%		T vs. A	0.65	1.12 (0.72–1.74)
***TRIB1*** **rs17321515**								
**genotype**								
AA	43	24.57%	16	28.07%		GG+AG vs. AA	0.60	0.84 (0.43–1.64)
AG	80	45.71%	29	50.88%	0.445	GG vs. AG+AA	0.24	0.63 (0.31–1.29)
GG	52	29.72%	12	21.05%		GG vs. AA	0.29	0.62 (0.27–1.45)
						AG vs. AA	1.00	0.97 (0.48–1.99)
						GG vs. AG	0.27	0.64 (0.30–1.36)
**Allele**								
A	166	47.43%	61	53.51%				
G	184	52.57%	53	46.49%		G vs. A	0.28	0.78 (0.51–1.20)
***TRIB1*** **rs2954029**								
**genotype**								
AA	45	25.71%	16	28.07%		TT+AT vs. AA	0.73	0.89 (0.45–1.73)
AT	80	45.72%	29	50.88%	0.547	TT vs. AT+AA	0.30	0.67 (0.33–1.37)
TT	50	28.57%	12	21.05%		TT vs. AA	0.40	0.68 (0.29–1.58)
						AT vs. AA	1.00	1.02 (0.50–2.08)
						TT vs. AT	0.35	0.66 (0.31–1.42)
**Allele**								
A	170	48.57%	61	53.51%				
T	180	51.43%	53	46.49%		T vs. A	0.39	0.82 (0.54–1.25)

^ χ^2^ test. * Fisher’s exact test.

**Table 4:** Distribution of the *PON1* rs662, rs854560 genotypes and alleles in unstable angina patients with and without arterial hypertension (HA).

	Without Arterial Hypertension (*n* = 87)		Arterial Hypertension (*n* = 145)		*p*-Value ^^^	Compared Genotypes or Alleles	*p*-Value *	OR (95% CI)
	n	%	n	%				
***PON1*** **rs662**								
**genotype**								
TT	41	47.13%	86	59.31%	0.143	CC+TC vs. TT	0.08	0.61 (0.36–1.05)
TC	40	45.98%	48	33.10%		CC vs. TC+TT	1.00	1.12 (0.40–3.15)
CC	6	6.89%	11	7.59%		CC vs. TT	0.79	0.87 (0.30–2.53)
						TC vs. TT	0.06	0.57 (0.33–1.00)
						CC vs. TC	0.60	1.53 (0.52–4.50)
**Allele**								
T	122	70.12%	220	75.86%				
C	52	29.88%	70	24.14%		C vs. T	0.19	0.75 (0.49–1.14)
***PON1*** **rs854560**								
**genotype**								
AA	43	49.43%	56	38.62%		TT+AT vs. AA	0.13	1.55 (0.91–2.66)
AT	36	41.38%	72	49.66%	0.271	TT vs. AT+AA	0.66	1.31 (0.54–3.18)
TT	8	9.19%	17	11.72%		TT vs. AA	0.37	1.63 (0.64–4.13)
						AT vs. AA	0.15	1.54 (0.87–2.70)
						TT vs. AT	1.00	1.06 (0.42–2.70)
**Allele**								
A	122	70.11%	184	63.45%				
T	52	29.89%	106	36.55%		T vs. A	0.16	1.35 (0.90–2.02)
***TRIB1*** **rs17321515**								
**genotype**								
AA	19	21.84%	40	27.59%	0.368	GG+AG vs. AA	0.35	0.73 (0.39–1.37)
AG	46	52.87%	63	43.45%		GG vs. AG+AA	0.65	1.21 (0.66–2.20)
GG	22	25.29%	42	28.96%		GG vs. AA	0.85	0.91 (0.43–1.92)
						AG vs. AA	0.25	0.65 (0.33–1.27)
						GG vs. AG	0.34	1.39 (0.74–2.65)
**Allele**								
A	84	48.28%	143	49.31%				
G	90	51.72%	147	50.69%		G vs. A	0.85	0.96 (0.66–1.40)
***TRIB1*** **rs2954029**								
**genotype**								
AA	20	22.99%	41	28.28%		TT+AT vs. AA	0.44	0.76 (0.41–1.40)
AT	46	52.87%	63	43.44%	0.376	TT vs. AT+AA	0.54	1.24 (0.67–2.28)
TT	21	24.14%	41	28.28%		TT vs. AA	1.00	0.95 (0.45–2.02)
						AT vs. AA	0.25	0.67 (0.35–1.29)
						TT vs. AT	0.33	1.43 (0.75–2.73)
**Allele**								
A	86	49.43%	145	50.00%				
T	88	50.57%	145	50.00%		T vs. A	0.92	0.98 (0.67–1.42)

^ χ^2^ test. * Fisher’s exact test.

**Table 5:** Associations between the analysed clinical parameters of patients with unstable angina and the *PON1* rs662 genotype.

Parameters	*PON1* rs662 Genotype										
		TT		TC		CC	TT vs. TC	CC vs. TC	TT vs. CC	CC+TC vs.	TT+TC vs. CC
										TT	
	*n*	Mean ± SD	*n*	Mean ± SD	*n*	Mean ± SD	*p*-Value ^&^				
Age (years)	127	61.71 ± 9.43	88	61.74 ± 9.94	17	66.41 ± 9.68	0.602	0.041	0.043	0.856	0.035
BMI (kg/m^2^)	127	28.58 ± 3.77	88	28.22 ± 4.39	17	27.59 ± 2.81	0.450	0.648	0.261	0.306	0.379
CH (mg/dL)	122	237.98± 60.40	85	220.99± 50.10	16	220.88 ± 47.00	0.037	0.985	0.286	0.030	0.512
HDL (mg/dL)	105	45.01 ± 8.37	70	44.51 ± 8.53	11	44.09 ± 8.57	0.785	0.869	0.644	0.703	0.723
LDL (mg/dL)	105	168.73 ± 51.33	70	156.81 ± 47.44	11	159.55 ± 60.22	0.184	0.815	0.327	0.138	0.483
TG (mg/dL)	121	145.27 ± 74.21	85	133.24 ± 76.08	16	132.88 ± 46.19	0.170	0.555	0.666	0.177	0.998

^&^—Mann–Whitney U test; BMI—body mass index; CH—total cholesterol in serum; HDL—high-density cholesterol in serum; LDL—low-density cholesterol in serum; TG—triacylglycerols in serum.

**Table 6:** Associations between the analysed clinical parameters of patients with unstable angina and the *PON1* rs854560 genotype.

Parameters	*PON1* rs854560 Genotype							
	AA	AT	TT	AA vs. AT	TT vs. AT	AA vs. TT	TT+AT vs.	AA+AT vs. TT
							AA	
	Mean ± SD	Mean ± SD	Mean ± SD	*p*-Value ^&^				
BMI (kg/m^2^)	28.03 ± 3.98	28.46 ± 3.76	29.32 ± 4.61	0.364	0.388	0.177	0.229	0.246
CH (mg/dL)	224.38 ± 47.78	227.51 ± 57.66	265.75 ± 69.52	0.971	0.005	0.004	0.342	0.003
HDL (mg/dL)	44.80 ± 8.37	44.21 ± 8.64	46.63 ± 7.73	0.660	0.131	0.221	0.945	0.144
LDL (mg/dL)	158.22 ± 47.09	161.43 ± 49.53	189.63 ± 58.46	0.704	0.031	0.020	0.257	0.017
TG (mg/dL)	142.19 ± 75.85	133.62 ± 70.15	156.21 ± 75.91	0.386	0.104	0.250	0.714	0.141

^&^—Mann–Whitney U test; BMI—body mass index; CH—total cholesterol in serum; HDL—high-density cholesterol in serum; LDL—low-density cholesterol in serum; TG—triacylglycerols in serum.

**Table 7:** Associations between the analysed clinical parameters of patients with unstable angina and the *TRIB1* rs17321515genotype.

Parameters	*TRIB1* r17321515 Genotype							
	AA	AG	GG	AA vs. AG	GG vs. AG	AA vs. GG	GG+AG vs.	AA+AG vs. GG
							AA	
	Mean ± SD	Mean ± SD	Mean ± SD	*p*-Value ^&^				
BMI (kg/m^2^)	28.34 ± 4.09	28.46 ± 3.99	28.25 ± 3.83	0.989	0.784	0.783	0.895	0.756
CH (mg/dL)	229.60± 47.51	229.11± 56.73	232.84 ± 63.24	0.616	0.711	0.950	0.714	0.821
HDL (mg/dL)	43.58 ± 7.25	43.63 ± 8.09	47.75 ± 9.27	0.841	0.010	0.023	0.378	0.005
LDL (mg/dL)	162.79 ± 42.39	163.32 ± 50.57	165.17 ± 57.56	0.818	0.864	0.997	0.879	0.902
TG (mg/dL)	132.05 ± 55.69	146.69 ± 84.69	135.36 ± 66.81	0.484	0.389	0.994	0.642	0.548

^&^—Mann–Whitney U test; BMI—body mass index; CH—total cholesterol in serum; HDL—high-density cholesterol in serum; LDL—low-density cholesterol in serum; TG—triacylglycerols in serum.

**Table 8:** Associations between the clinical parameters of patients with unstable angina and the *TRIB1* rs2954029 genotype.

Parameters	*TRIB1* rs2954029 Genotype							
	AA	AT	TT	AA vs. AT	TT vs. AT	AA vs. TT	TT+AT vs.	AA+AT vs. TT
							AA	
	Mean ± SD	Mean ± SD	Mean ± SD	*p*-Value ^&^				
BMI (kg/m^2^)	28.26 ± 4.06	28.47 ± 3.98	28.31 ± 3.87	0.893	0.856	0.934	0.957	0.871
CH (mg/dL)	230.78 ± 47.48	229.79 ± 57.21	230.60 ± 63.06	0.556	0.990	0.635	0.540	0.840
HDL (mg/dL)	43.84 ± 7.44	43.51 ± 7.92	47.88 ± 9.42	0.688	0.007	0.030	0.494	0.005
LDL (mg/dL)	164.10 ± 42.03	164.13 ± 51.42	162.58 ± 57.04	0.727	0.856	0.652	0.661	0.746
TG (mg/dL)	133.22 ± 55.08	146.75 ± 84.89	134.22 ± 67.22	0.578	0.332	0.748	0.815	0.420

^&^—Mann–Whitney U test; BMI—body mass index; CH—total cholesterol in serum; HDL—high-density cholesterol in serum; LDL—low-density cholesterol in serum; TG—triacylglycerols in serum.

## Data Availability

The data that support the findings of this study, except for patients’ identifiers, are available from the corresponding author upon reasonable request.
